# Optimal timing of viral load monitoring during pregnancy to predict viraemia at delivery in HIV‐infected women initiating ART in South Africa: a simulation study

**DOI:** 10.1002/jia2.25000

**Published:** 2017-11-24

**Authors:** Maia Lesosky, Tracy Glass, Elton Mukonda, Nei‐Yuan Hsiao, Elaine J. Abrams, Landon Myer

**Affiliations:** ^1^ Division of Epidemiology & Biostatistics School of Public Health & Family Medicine University of Cape Town Cape Town South Africa; ^2^ National Health Laboratory Services Cape Town South Africa; ^3^ Division of Medical Virology Faculty of Health Sciences University of Cape Town Cape Town South Africa; ^4^ ICAP Mailman School of Public Health Columbia University New York NY USA; ^5^ College of Physicians & Surgeons Columbia University New York NY USA; ^6^ Centre for Infectious Disease Epidemiology & Research School of Public Health & Family Medicine University of Cape Town Cape Town South Africa

**Keywords:** HIV, antiretroviral therapy, viral load monitoring, pregnancy, mathematical model, simulation

## Abstract

**Introduction:**

HIV viral load (VL) monitoring is a central tool to evaluate ART effectiveness and transmission risk. There is a global movement to expand VL monitoring following recent recommendations from the World Health Organization (WHO), but there has been little research into VL monitoring in pregnant women. We investigated one important question in this area: when and how frequently VL should be monitored in women initiating ART during pregnancy to predict VL at the time of delivery in a simulated South African population.

**Methods:**

We developed a mathematical model simulating VL from conception through delivery using VL data from the Maternal and Child Health – Antiretroviral Therapy (MCH‐ART) cohort. VL was modelled based on three major compartments: pre‐ART VL, viral decay immediately after ART initiation and viral maintenance (including viral suppression and viraemic episodes). Using this simulation, we examined the performance of various VL monitoring schema in predicting elevated VL at delivery.

**Results and discussion:**

If WHO guidelines for non‐pregnant adults were used, the majority of HIV‐infected pregnant women (69%) would not receive a VL test during pregnancy. Most models that based VL monitoring in pregnancy on the time elapsed since ART initiation (regardless of gestation) performed poorly (sensitivity <50%); models that based VL measures in pregnancy on the woman's gestation (regardless of time on ART) appeared to perform better overall (sensitivity >60%). Across all permutations, inclusion of pre‐ART VL values had a negligible impact on predictive performance (improving test sensitivity and specificity <6%). Performance of VL monitoring in predicting VL at delivery generally improved at later gestations, with the best performing option a single VL measure at 36 weeks’ gestation.

**Conclusions:**

Development and evaluation of a novel simulation model suggests that strategies to measure VL relative to gestational age may be more useful than strategies relative to duration on ART, in women initiating ART during pregnancy, supporting better integration of maternal and HIV health services. Testing turnaround times require careful consideration, and point‐of‐care VL testing may be the best approach for measuring VL at delivery. Broadening the scope of this simulation model in the light of current scale up of VL monitoring in high burden countries is important.

## 1 Introduction

There are more than 18 million HIV‐infected women of childbearing age globally and an estimated 1.4 million pregnancies annually in HIV‐infected women 1. Viral suppression through the use of lifelong antiretroviral therapy (ART) is the critical intervention to support the long‐term health of HIV‐infected women and mothers and the prevention of both sexual and mother‐to‐child transmission (MTCT). While there have been global advances in programmes that promote universal initiation of lifelong ART for PMTCT 2, major concerns have emerged related to postpartum ART adherence 3, and up to one‐third of women initiating ART in pregnancy experience a loss of viral control during the postpartum period 4.

HIV viral load (VL) monitoring is the central tool to evaluate ART effectiveness and transmission risk, and there is a global movement to expand use of VL monitoring following on recent recommendations from the World Health Organisation (WHO) 5, 6. While there are well‐developed guidelines for VL monitoring in non‐pregnant adults on ART, there has been little consideration given to implementation of VL monitoring in pregnant and postpartum women. Despite the importance of effective ART services during this period, current guidelines for adult VL monitoring in most countries do not address pregnant and postpartum women specifically. Although South African guidelines 7 have recommendations specific to pregnant and postpartum women, there is little empirical evidence to support this approach and the generalisability to other settings is unclear. In turn, there is a clear and urgent need for research to guide evidence‐based recommendations into optimal VL monitoring strategies during pregnancy and breastfeeding.

Mathematical simulations are ideally suited to explore diverse scenarios for monitoring disease progression and/or response to treatment. In many contexts, designing empirical studies that examine different disease monitoring strategies can be impossible due to prohibitive study duration, logistics, ethical considerations and/or research costs 8, 9, 10, 11. While limited aspects of VL monitoring strategies have been investigated in recent modelling work 12, these have not included the key population of pregnant and breastfeeding women.

There are basic questions facing country programmes and international guidelines around when and how frequently VL should be monitored in HIV‐infected women initiating ART during pregnancy. Recent WHO guidance notes that an enhanced regimen of antiretroviral prophylaxis may be given to newborns of women with a raised VL at delivery, and the period of labour and delivery is well‐recognised as a high‐risk window for MTCT 1. However, the best approach to predict VL at delivery among women initiating ART in pregnancy has not been explored. To help address this issue, we used a simulation based on South African data to examine the ability of VL monitoring at different time points in pregnancy to predict VL at the time of delivery.

## 2 Methods

Working in R (R Foundation, Vienna, Austria), we developed a simulation of VL from conception through delivery, and then examined the performance of various VL monitoring schema in predicting VL at delivery. This simulation approach models VL at regular intervals before, during and after ART initiation in pregnancy, providing insights that would not be possible through direct observation of patients. For this, a cohort of HIV‐infected women not using ART at the time of conception was simulated on a weekly time step from conception through delivery. The model tracked VL (including VL pre‐ART and after ART initiation), timing of ART initiation in pregnancy, the presence and timing of (i) initial viral suppression and (ii) elevated VL after ART initiation, and the date and gestation of delivery.

### 2.1 Simulation model

**Figure 1:**
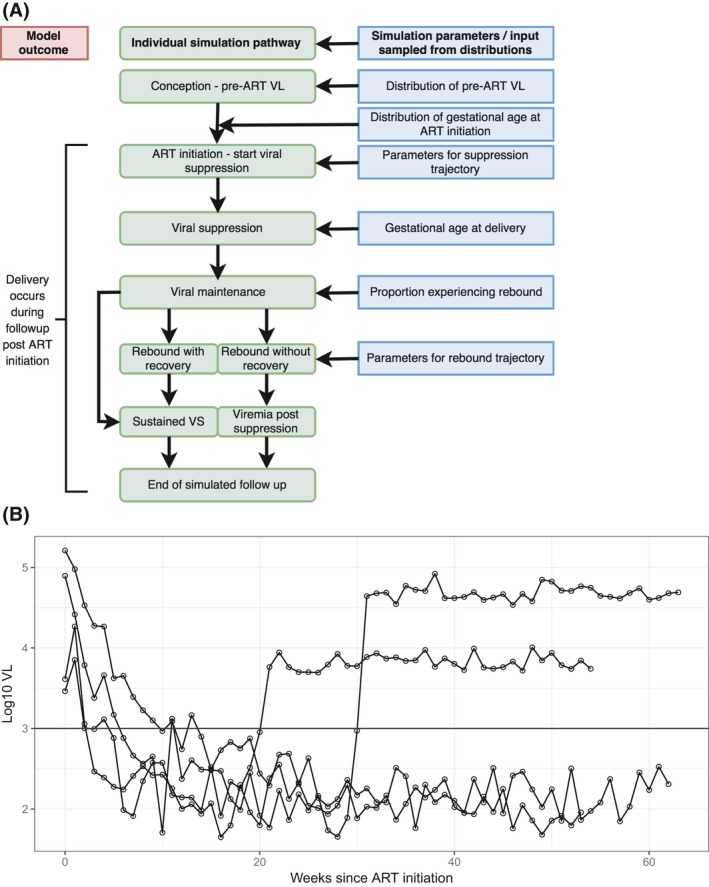
Model structure (**A) and sample output (B) of viral load trajectories for simulated cohort for women used in the study. Figure (A) shows the simplified schematic of the model used to simulate viral loads in HIV+ pregnant women initiating ART. Figure (B) shows a plot of random sample of trajectories for four individuals in the viral load simulation; for illustrative purposes this sample purposefully selects two patients who achieve and maintain viral suppression, and two patients who experience elevated viral load after initial suppression.**

Using this model structure, we simulated continuous VL measures in 10,000 South African women initiating ART in pregnancy. Figure 1A shows a schematic for the model structure. The model was parameterised using data from the Maternal and Child Health – Antiretroviral Therapy (MCH‐ART) study (ClinicalTrials.gov register number NCT01933477) 13. This study followed a cohort of HIV‐infected women from the start of antenatal care, including 620 women initiating ART during pregnancy who underwent regular viral load testing, as described previously 5, 14, 15. Parameters drawn on from MCH‐ART were gestational age at ART initiation (weeks), gestational age at delivery (weeks), viral suppression trajectories (estimated in copies/mL with fractional polynomials), rate of viraemic episodes after initial viral suppression (as a proportion with VL ≥1000 copies/mL over N eligible) and outcomes after initial viral rebound (ongoing viraemia or viral resuppression (as a proportion). Of the individuals that experienced viraemic episodes after initial suppression, a fraction experienced complete loss of viral control, with their subsequent VL sampled from the woman's pre‐ART VL distribution. Those that lost viral control only temporarily (“viral blips”) regained viral suppression and remained virally suppressed for the remainder of the observation period. The duration of viral blips varied, depending on the magnitude of the response and the modelled VL trajectory. Fractional polynomial models were used to estimate trajectories on both sides of the maximum magnitude of the viral blip. Median and interquartile range (IQR) are calculated from the source data for continuous measures and frequency (percent) for counts.

### 2.2 VL trajectory modelling

Continuous valued VL was simulated for each woman at each week of pregnancy. VL was modelled based on three major compartments: pre‐ART VL, viral decay immediately after ART initiation and viral maintenance (including viral suppression and viraemic episodes). Each compartment had a different generation model and each simulated woman transitioned independently of other individuals through all conditions. VL measures were sampled from different distributions per compartment, and have dependencies on that woman's pre‐ART VL value. Figure 1B shows sample VL distributions generated by the simulation. The slopes for suppression and rebound trajectories were based on fractional polynomial models, the parameters of which were dependent on pre‐ART VL values. Throughout, VL was simulated as a continuous measure and additive non‐Gaussian noise was included in all simulations.

### 2.3 Viral load monitoring strategies

Using this simulation, we evaluated the predictive performance of different approaches to VL monitoring at different time points during the antenatal period to predict VL at the time of delivery. Given the costs of VL testing, we focused on strategies which minimized the number of VL tests required for a woman. Three broad approaches to monitoring were examined: (i) a single VL test conducted based on duration of ART use (regardless of gestation), (ii) a single VL test based on gestation (regardless of duration of ART use) and (iii) the addition of a pre‐ART VL test to assist in either (i) or (ii). For approach (i), we investigated the results of VL testing at 4, 8, 12, 16, 20 and 24 weeks after ART initiation, and for (ii) we examined testing at 12, 20, 24, 32 and 36 weeks gestational age. For all analyses, women in the simulated cohort were eligible if they had initiated ART by the time of the proposed test (the input distribution based on the distribution of gestations at ART initiation in the MCH‐ART data) and had not delivered by that time (based on the distributions of gestations at delivery in the MCH‐ART data 15). In all cases, we describe the proportion of women who would not be tested under each strategy due to either of these factors (e.g. late ART initiation or premature delivery).

Different classes of predictive models were applied to simulated data to predict continuous VL at delivery. Predicted VL at delivery was made discrete and utilised for evaluation of model performance as a binary construct of <1000 versus ≥1000 copies/mL in keeping with WHO guidelines and based on the finding that MTCT transmission risks are greatly increased above 1000 copies/mL 16, 17. Models were applied to the full cohort of 10,000 and to a subset of individuals initiating ART before 20 weeks gestational age (early ART initiation). Simple linear models were examined; here, we present estimates based on a last observation carried forward (LOCF) model as it represents the most common approach in real‐world clinical care; this model assumes the VL at delivery will be equal to the VL measures during gestation (i.e. the VL measure is “carried forward” to delivery). Linear regression models were used to incorporate pre‐ART VL into the LOCF model.

### 2.4 Model performance outputs

Model parameters are summarised with median (IQR) for continuous measures and percent (standard deviation) for binary measures. For each of the specified VL monitoring time points, the sensitivity (SE), specificity (SP), negative likelihood ratio (LR−), positive likelihood ratio (LR+), likelihood ratio (LR+/LR−), positive (PPV) and negative predictive value (NPV) were calculated evaluating the ability of the categorised VL measured at that point in pregnancy to predict VL at the time of delivery, and reported with estimated 95% confidence intervals. Predictive models were run independently on the “training” simulation run of 10,000 individuals, performance was evaluated on a “test” simulation run, again of 10,000 individuals; initiated with a different random seed.

## 3 Results

### 3.1 Model calibration

Table 1 shows key features of the simulated cohort. Averaged across runs, the median (IQR) gestational age at ART initiation in the simulated cohort was 18 weeks (14, 23) and pre‐ART VL was 3.99 log_10_ copies/mL (3.28, 4.66). The mean percent of women with VL <1000 copies/mL at the time of delivery was 89% (sd, 0.3%) and median (IQR) time on ART at delivery was 18 weeks (12, 23). The median (IQR) gestation at delivery was 39 weeks (38, 40).

**Table 1:** Summary statistics from simulation model of viral load monitoring at specified time points relative to duration on ART or relative to gestational age. Women were eligible for testing if they had initiated ART in pregnancy and had not delivered at the time point of evaluation

	VL monitoring time point (number of weeks on ART)						VL monitoring time point (gestational age (weeks))					
	4	8	12	16	20	24	12	20	24	32	36	Delivery
N eligible	9724	9112	8104	6906	5106	3082	1406	5047	6980	8946	9135	10,000
Percent eligible	97.2	91.1	81.0	69.1	51.1	30.8	14.1	50.5	69.8	89.5	91.4	100
Median (IQR) gestational age at time of VL test	24 (20, 29)	28 (23, 32)	31 (27, 35)	34 (30, 37)	36 (32, 39)	38 (34, 40)	‐	‐	‐	‐	‐	
Median (IQR) weeks on ART at time of VL test	‐	‐	‐	‐	‐	‐	3 (1, 5)	4 (2, 8)	6 (3, 10)	12 (8, 16)	16 (11, 20)	18 (12, 23)
Percent of women who have initiated ART by this time	‐	‐	‐	‐	‐	‐	14.1	50.5	69.8	89.2	91.5	97.9

### 3.2 Statistical predictive models

On each analysis set, statistical models were applied using the VL measure, time on ART and pre‐ART VL to develop a model for VL at delivery (training data). The details of the models can be found in Table 2. Each model was applied to a new simulation run (holdout data), and the model performance statistics were calculated based on correct model predicted viraemia at delivery or not (based on ≥1000 copies/mL).

**Table 2:** Predictive model performance statistics resulting from last observation carried forward model of viral load monitoring at specified time points. Women were eligible for testing if they had initiated ART in pregnancy and had not delivered at the time point of evaluation

Timing of test	n	% of women eligible for testing	Sensitivity (95% CI)	Specificity (95% CI)	LR test (LR+/LR‐)	LR positive (95% CI)	LR negative (95% CI)	Negative predictive value (95% CI)	Positive predictive value (95% CI)
Testing based on duration of ART use									
Four weeks on ART	9724	1	0.74 (0.71, 0.77)	0.64 (0.63, 0.65)	5.05	2.05 (1.96, 2.15)	0.41 (0.36, 0.45)	0.96 (0.96, 0.97)	0.17 (0.16, 0.18)
Eight weeks on ART	9112	0.9	0.5 (0.46, 0.54)	0.86 (0.85, 0.87)	6.19	3.6 (3.27, 3.96)	0.58 (0.54, 0.63)	0.96 (0.95, 0.96)	0.21 (0.19, 0.23)
12 weeks on ART	8104	0.8	0.36 (0.31, 0.41)	0.95 (0.94, 0.95)	10.0	6.75 (5.74, 7.93)	0.67 (0.63, 0.73)	0.97 (0.96, 0.97)	0.26 (0.22, 0.29)
16 weeks on ART	6906	0.7	0.38 (0.32, 0.44)	0.97 (0.97, 0.98)	23.59	15.06 (12.19, 18.6)	0.64 (0.58, 0.7)	0.97 (0.97, 0.98)	0.39 (0.34, 0.45)
20 weeks on ART	5106	0.5	0.42 (0.35, 0.5)	0.99 (0.99, 0.99)	72.27	42.3 (30.47, 58.74)	0.59 (0.52, 0.66)	0.98 (0.98, 0.98)	0.60 (0.51, 0.69)
24 weeks on ART	3082	0.4	0.62 (0.52, 0.72)	0.99 (0.99, 1.00)	290.29	109.5 (66.6, 179.9)	0.38 (0.29, 0.48)	0.99 (0.98, 0.99)	0.79 (0.69, 0.87)
Testing based on gestational age									
12 weeks’ gestation	1406	0.1	0.77 (0.61, 0.88)	0.43 (0.4, 0.46)	2.48	1.34 (1.13, 1.6)	0.54 (0.31, 0.94)	0.98 (0.97, 0.99)	0.04 (0.03, 0.06)
20 weeks’ gestation	5047	0.5	0.56 (0.48, 0.63)	0.57 (0.56, 0.59)	1.67	1.3 (1.13, 1.49)	0.78 (0.66, 0.92)	0.97 (0.97, 0.98)	0.04 (0.04, 0.05)
24 weeks’ gestation	6980	0.7	0.58 (0.53, 0.64)	0.69 (0.68, 0.7)	3.13	1.89 (1.7, 2.08)	0.6 (0.53, 0.69)	0.97 (0.97, 0.98)	0.08 (0.07, 0.09)
32 weeks’ gestation	8946	0.9	0.58 (0.54, 0.61)	0.88 (0.87, 0.89)	9.98	4.81 (4.4, 5.25)	0.48 (0.44, 0.53)	0.96 (0.96, 0.97)	0.27 (0.24, 0.29)
36 weeks’ gestation	9118	0.9	0.72 (0.69, 0.75)	0.96 (0.95, 0.96)	56.9	16.67 (14.94, 18.6)	0.29 (0.26, 0.33)	0.97 (0.97, 0.97)	0.63 (0.60, 0.66)

Most models that based VL monitoring in pregnancy on the time elapsed since ART initiation (regardless of gestation) demonstrated poor sensitivity (SE <50%) and good specificity (>85%) (Table 2). When monitoring in pregnancy was based on time since ART initiation, the optimal timing for a single VL appeared to be a VL measured at 20 weeks after ART initiation (SE: 42%, SP: 99%); however, only 50% of women would be eligible for this measure (the remainder having delivered by this time point). Generally, these models incorrectly specified a relatively small proportion of individuals as suppressed when they were truly viraemic at delivery, but misclassified a much higher proportion of women as being viraemic at delivery when they were truly suppressed; this was due in large part to the inclusion of women who initiated ART late in pregnancy and had not yet achieved initial viral suppression by the time of testing. If VL monitoring was based on guidelines for non‐pregnant adults, with a first VL conducted 6 months after ART initiation, only 31% of the cohort would be eligible to be tested before delivery.

**Figure 2:**
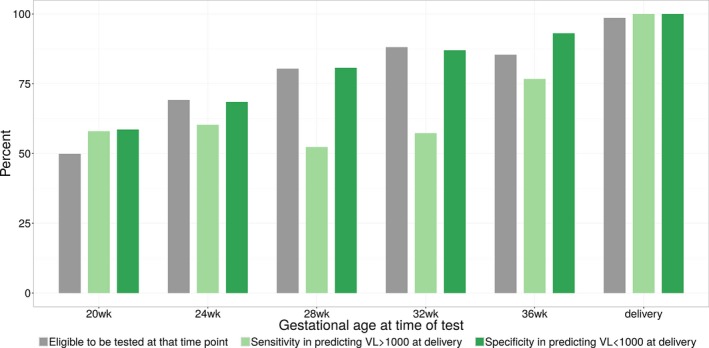
Sensitivity and specificity of viral load monitoring conducted at selected gestations during pregnancy to detect viral load ≥1000 copies/mL at the time of delivery.

In contrast, most models that based VL measures in pregnancy on the woman's gestation (regardless of time on ART) appeared to perform better overall (Table 2). VL tests conducted late in pregnancy appeared able to test higher proportions of women in the simulated cohort. Model performance generally improved at later gestations, with perfect sensitivity and specificity achieved by VL testing at the time of delivery, by definition. In addition, the proportion of the cohort eligible to be tested decreased late in the third trimester as premature deliveries pre‐empted VL testing in pregnancy. Overall, the optimal time point appeared to be testing at 36 weeks’ gestation with approximately 90% of women eligible to be tested and relatively high sensitivity (72%) and specificity (95%) observed in detecting VL ≥1000 copies/mL at delivery (Figure 2).

Across all permutations, inclusion of pre‐ART VL values had a negligible impact on predictive performance when evaluating VL monitoring based on either gestational age or duration on ART, using linear models. Models which included pre‐ART VL in addition to a VL after ART initiation increased modelled specificities and sensitivities by <6%, compared to the corresponding models without pre‐ART VL, however, were hampered by a tendency to make out of range predictions due to the linear structure.

## 4 Discussion

This simulation study provides several important new insights into routine VL monitoring strategies for women who initiate ART during pregnancy. First, if monitoring in pregnancy is based on current guidelines for non‐pregnant adults, with a first VL after 6 months on ART, only 31% of women in this simulation would be tested in pregnancy. Second, VL monitoring strategies based on time on ART may not be ideal for VL monitoring in pregnancy, while the best‐performing monitoring schedule in pregnancy appears to be a single test at 36 weeks’ gestation. Third, the addition of pre‐ART VL measures improves prediction only by a small proportion of those with elevated VL at delivery, and may not be a cost‐effective approach to VL monitoring in pregnancy.

Monitoring strategies based on gestational age verses time on ART may be easier to implement in many settings, as they could coincide with routine antenatal visits. We found that a single VL test at 36 weeks’ gestation can predict 73% of the 9% of women with VL ≥1000 copies/mL at delivery. This is reassuring as this approach is implied by recent WHO guidelines 5, however, we found that only 91% of all HIV‐infected women would be tested at this time due to preterm deliveries, and in turn testing at later gestations (such as 37 or 38 weeks’ gestation) would increase the proportion of women who could be tested towards 100%. By definition, the optimal approach to predicting VL at delivery would be to test VL at the time of delivery. However, testing turnaround times for existing VL monitoring systems (which are routinely >1 week and often >4 weeks in many parts of sub‐Saharan Africa, from the time of specimen collection to the time of result return) would preclude VL testing at or just prior to delivery from informing infant management immediately postpartum, including the initiation of enhanced antiretroviral prophylaxis 18. To help address this issue, point‐of‐care (POC) VL tests conducted at delivery could make VL results available for patient management within hours of specimen collection 19, 20, and would theoretically have perfect sensitivity and specificity in predicting VL at delivery, in addition to being possible to conduct on close to 100% of women delivering (Figure 2).

There are several limitations in this model‐based analysis. The simulation is based on parameters from a single South African cohort and validation with other datasets from other settings is required, noting that data on VL trajectories in HIV‐infected women initiating ART in pregnancy in low‐resource settings are limited. We did not include in our model the turnaround times associated with VL monitoring, and thus this work assumes that all VL specimens collected would have results available; given the complexities of the VL “cascade” in many LMIC settings 21, the implications of different turnaround times for interpreting these findings should be considered carefully. And finally, we did not consider the costs of testing, or the subsequent cost‐effectiveness of different VL monitoring approaches, noting that these are critical considerations for policymaking. Broadening the consideration of VL monitoring to include the possible role of VL monitoring in supporting ART adherence may enhance the cost‐effectiveness of monitoring, but data to support this are limited 22.

More broadly, this work demonstrates the value of simulation studies for investigating complex questions related to the implementation of VL monitoring in LMIC settings. While we focused on women initiating ART in pregnancy, there is also a growing population of women who enter antenatal care already on ART (having initiated before pregnancy), and the optimal VL monitoring strategies for this population require further attention in similar modelling approaches 15. These methods can also be applied to address a wider range of issues, including VL monitoring during breastfeeding, a time of growing concern for MTCT risk 1, or in other patient populations. With expanding insights into the implementation and findings of routine VL monitoring in countries where HIV is prevalent, there is a growing body of data to help inform the design and parameterization of such simulations, and this is an important area for future investigation.

In summary, this simulation suggests that pregnant women warrant VL monitoring approaches different from non‐pregnant adults. A single VL test conducted late in gestation may be used to predict approximately three‐quarters of all elevated VL at delivery, but effective implementation would require rapid turnaround times. Furthermore, POC VL testing may be important to detect larger proportions of viraemic women on ART for intervention.

## Competing interests

None of the authors have any competing interests to declare.

## Authors’ contributions

ML, LM and EA developed the initial concept and idea. ML developed and wrote the first version of the simulation models. NYH assisted in study conceptualization provided expertise regarding viral load monitoring. ML, TG and EM carried out the simulations, calibration and statistical analysis. ML drafted the first version of the manuscript. All authors contributed to writing and reviewing the science. All authors have read and approved the final manuscript.
